# Complete Remission of Acute Myeloid Leukemia following Cisplatin Based Concurrent Therapy with Radiation for Squamous Cell Laryngeal Cancer

**DOI:** 10.1155/2016/8581421

**Published:** 2016-04-03

**Authors:** Mayur D. Mody, Harpaul S. Gill, Kristin A. Higgins, Nabil F. Saba, Vamsi K. Kota

**Affiliations:** ^1^Department of Internal Medicine, Emory University School of Medicine, Atlanta, GA 30322, USA; ^2^Department of Radiation Oncology, Emory University, Atlanta, GA 30322, USA; ^3^Winship Cancer Institute of Emory University, Atlanta, GA 30322, USA; ^4^Department of Hematology and Medical Oncology, Emory University, Atlanta, GA 30322, USA

## Abstract

Acute myeloid leukemia (AML) is a myeloid disorder with several established treatment regimens depending on patient and leukemic factors. Cisplatin is known to have strong leukemogenic potential and is rarely used even as salvage therapy in relapsed or refractory AML. We present a patient simultaneously diagnosed with AML and squamous cell carcinoma of the larynx, who was found to be in complete remission from AML following treatment with cisplatin based chemoradiotherapy for his laryngeal cancer.

## 1. Introduction

Acute myeloid leukemia (AML) with a normal karyotype composes the single largest cytogenetic group of AML and is estimated to account for 45% of adults with de novo AML [1]. For older adults with favorable or intermediate risk AML and an ECOG performance status of two or less and few comorbidities, induction chemotherapy is recommended with a combination of an anthracycline and cytarabine. However, if older patients are determined to have indolent AML, severe comorbidities, or unfavorable risk disease, less intensive chemotherapy with DNA hypomethylating agents (azacitidine and decitabine) or low-dose cytarabine is favored compared to conventional remission induction chemotherapy [2]. We present the case of a patient found to be in complete remission from AML following treatment with cisplatin based chemoradiotherapy for squamous cell carcinoma of the larynx. This patient's clinical course demonstrates a peculiar case where cisplatin served as a therapeutic agent for AML.

## 2. Case Description

**Figure 1:**
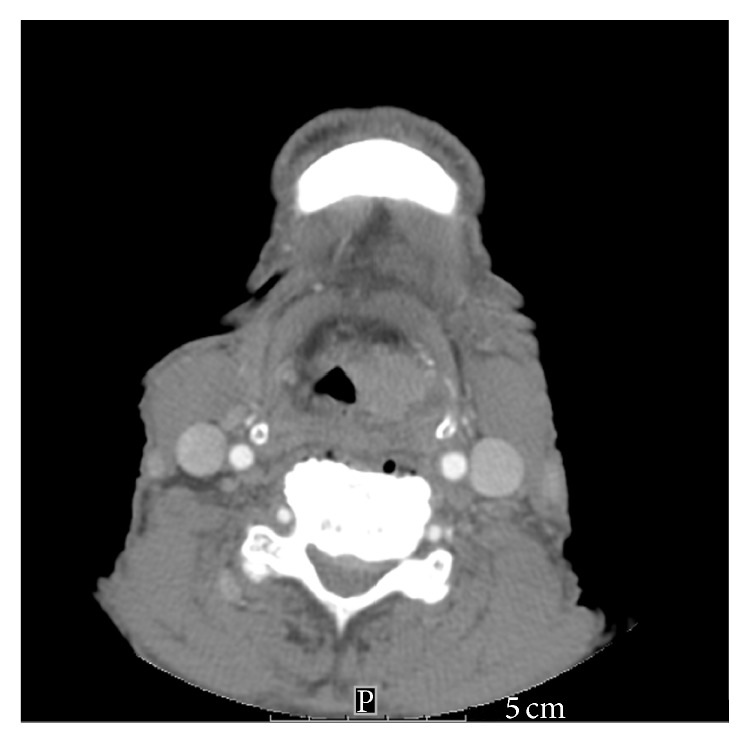
Neck CT showing patient's left glottis tumor.

A 68-year-old male presented to an NCI-designated Cancer Center for evaluation of simultaneously diagnosed laryngeal cancer and AML during a hospitalization at an outside hospital (OSH). The patient presented to this OSH with progressive throat discomfort, hoarseness, stridor, and shortness of breath over several months. The patient endorsed a seventy-pack-year smoking history as well as chronic alcohol use. A neck CT was obtained and revealed a 2.2 × 1.7 × 1.8 cm left glottis tumor that extended from the supraglottis down to the subglottis (Figure 1). There was no evidence of adenopathy on the scan. Biopsy obtained showed squamous cell carcinoma (SCC), p16 negative. Upon staging, a diagnosis of Stage III T3N1M0 squamous cell carcinoma of the supraglottis was made.

During this hospitalization, the patient was also found to be pancytopenic (Table 1).

A bone marrow biopsy was obtained which revealed AML with myelodysplasia-related changes and 30% myeloblasts. Cytogenetic evaluation was unremarkable for abnormalities and a mutational analysis was not done. Upon improvement of his clinical condition, the patient was discharged with follow-up arranged at an NCI-designated Cancer Center, where he was evaluated by a leukemia specialist, head and neck oncologist, radiation oncologist, and otolaryngologist. Regarding the patient's SCC, his profound level of neutropenia in the setting of AML precluded him from total laryngectomy with neck dissection. Chemotherapy with a platinum based agent along with concurrent radiation therapy was recommended. Given the patient's age and performance status (ECOG 2), he was determined not to be a candidate for intensive treatment for his AML with induction therapy. As the patient was symptomatic from his head and neck cancer, a decision was made to proceed with treatment of his SCC first. However, it was noted that holding off AML treatment until the patient completed and recovered from concurrent chemoradiation for his SCC would not be feasible, as the AML would most likely be fatal in the interim period. Therefore, a very low dose of decitabine was initially proposed for his AML while the patient was undergoing treatment for his SCC.

However, chemoradiation therapy was initially delayed as patient developed complications from his malignancies, including a hospitalization for pneumonia. Of note, hospice care had been recommended during this hospitalization due to the belief that the patient would not tolerate curative treatment for his locally advanced head and neck cancer.

The patient was eventually started on chemoradiation two months following his initial evaluation. Given the complications prior to initiation of chemoradiation for his SCC, decitabine was never administered. He was found to have low platelets (56 K/uL) prior to initiation of chemotherapy and, as a result, was started on weekly cisplatin at 20 mg/m^2^. He completed 5 weeks of weekly cisplatin therapy, with the dose being increased to 30 mg/m^2^ after a rise in platelets (80 K/uL) after the first week of therapy. The patient also successfully completed radiation therapy, with a total of 70 Gy given to the gross supraglottic disease and the involved left level II/III lymph nodes.

The patient tolerated concurrent therapy well, with no lapses in treatment or transfusions needed. A follow-up PET obtained approximately 3 months after treatment demonstrated a complete response with no FDG activity to suggest residual disease. A repeat bone marrow biopsy was recommended to establish present condition of patient's AML, with plan to start patient on hypomethylating agents at varying doses pending bone marrow results. Bone marrow biopsy results showed a normocellular marrow with trilineal hematopoiesis, with blasts comprising less than 5% of the nucleated cells on the aspirate smear. Phenotypic analysis via flow cytometry failed to identify any abnormal hematolymphoid cell populations. The patient remains in complete hematological remission approximately one year from initial diagnosis and eight months from completion of chemoradiation.

## 3. Discussion

In the context of AML, cisplatin, among other platinum based agents, has been implicated as cytotoxic agent that has strong leukemogenic potential and puts patients at risk for developing therapy-related myeloid neoplasms [3]. In an analytical study of 18.657 testicular cancer patients by Travis et al., it was shown that a cumulative exposure of 650 mg cisplatin/m^2^ for testicular cancer treatment increased the relative risk of leukemia in these patients by 3.2-fold, while larger doses (1000 mg cisplatin/m^2^) were linked with sixfold increase in relative risk [4]. Cisplatin's carcinogenic potential is thought to be augmented when combined with other carcinogenic therapies, such as radiation in the setting of concurrent therapy regimens [5].

Cisplatin's role in the therapy for AML, on the other hand, is much less understood. Cisplatin has been previously considered as combination chemotherapy in relapsed or refractory AML. In a phase I trial by Seiter et al., five of 20 patients (15 of which had AML) demonstrated a significant reduction in bone marrow blasts, as cisplatin was thought to increase the sensitivity of leukemia cells to temozolomide by depleting MGMT [6]. Similarly, Lee et al. showed the combination of high dose cytarabine, etoposide, and cisplatin to be effective salvage chemotherapy in high-risk relapsed or refractory AML, with overall complete remission rate of 31% among 49 patients [7].

Cisplatin is a DNA-damaging agent that is widely used in cancer chemotherapy. Cisplatin has significant activity in solid tumor malignancies, with successful therapeutic outcomes for head and neck, lung, ovarian, and testicular cancers [8]. The traditionally accepted mechanism of action of cisplatin involves its cross-linking to DNA, forming intra- and interstrand adducts, which unwind the duplex and attract high-mobility-group domain and other proteins. The shielding effect of these proteins results in poor repair of the cisplatin-modified DNA, thereby leading to activation of several signaling transduction pathways (including those involving ATF, p53, p73, and MAPK) and ultimately cell apoptosis [9, 10]. However, our understanding of cisplatin-induced cell death remains limited, as it is a nonspecific drug that reacts not only with DNA, but also with proteins, resulting in several other proposed and studied mechanisms of action for cell death in addition to cell apoptosis [11]. Pestell et al. showed that populations of cisplatin treated cells were undergoing not only apoptosis, but also cell death via a necrotic route [12].

Furthermore, the idea of apoptosis and necrosis as being two distinct mechanisms of cellular death has been challenged, and scientists have instead proposed a continuum of cellular death, where a cell fall on this continuum depends on specific factors such as the availability of energy and metabolic condition of the cell [13]. Segal-Bendirdjian and Jacquemin-Sablon determined that cisplatin-induced cell death in L1210 leukemic cells was at least partly a result of an unfinished apoptotic program [14]. In addition, Perez proposed that, in addition to its DNA-damaging effects, cisplatin damages molecules involved in cellular energy supply (i.e., ATP) and also proteins involved in the apoptotic process (i.e., p53, Bax, Bcl-2, and caspases), leading to necrotic cell death [15].

Existing evidence suggests that approximately 1% of cellular cisplatin interacts with DNA and forms DNA adducts [11]. Further research on cisplatin's cytotoxic effect on leukemic cells, via apoptosis, necrosis, or a combination of both, is needed to better understand the potential therapeutic effect cisplatin had on our patient's AML.

## 4. Conclusion

A review of recent literature reveals several recent studies exploring cisplatin's cytotoxic effects on several forms of leukemic cells, including Bcr-Abl positive, acute lymphoblastic, and acute promyelocytic leukemic cells [9, 16, 17]. Any therapeutic potential of cisplatin in the setting of AML remains to be elucidated. To our knowledge, we describe the first documented case of complete remission from de novo AML seen in a patient treated with cisplatin as part of concurrent chemoradiation therapy for SCCHN.

## Figures and Tables

**Table 1:** Complete blood count from OSH.

WBC	HGB	HCT	PLT	Neutrophils	Lymphocytes	Monocytes	Eosinophils
K/uL	g/dL	%	K/uL	%	%	%	%
1.7	10.5	30.3	40	24	72	2	2
